# Chronic Critical Illness: Current Aspects of the Problem (Review)

**DOI:** 10.17691/stm2022.14.3.08

**Published:** 2022-05-28

**Authors:** A.L. Parfenov, V.P. Razzhivin, M.V. Petrova

**Affiliations:** Senior Researcher, Professor, Department of Anesthesiology and Intensive Care Medicine; Federal Research and Clinical Center of Intensive Care Medicine and Rehabilitology, 25 Petrovka St., Bld. 2, Moscow, 107031, Russia; Associate Professor, Professor, Department of Anesthesiology and Intensive Care Medicine; Federal Research and Clinical Center of Intensive Care Medicine and Rehabilitology, 25 Petrovka St., Bld. 2, Moscow, 107031, Russia; Professor, Deputy Director for Scientific and Clinical Work; Federal Research and Clinical Center of Intensive Care Medicine and Rehabilitology, 25 Petrovka St., Bld. 2, Moscow, 107031, Russia; Head of the Department of Anesthesiology and Intensive Care Medicine with the Course of Rehabilitation; Peoples’ Friendship University of Russia, 6 Miklukho-Maklaya St., Moscow, 117198, Russia

**Keywords:** chronic critical illness, persistent inflammation, persistent inflammation, immunodepression, and catabolic syndrome, post-intensive care syndrome, microbiome

## Abstract

Chronic resuscitation patients who have survived the acute phase of a disease represent a fast-growing cohort of patients requiring specialized medical assistant in intensive care and resuscitation units (ICRU) for several months or years. The term “chronic critical illness” (CCI) was proposed for such patients in the mid-80s of the last century. Patients with CCI make up from 5 to 20% of ICRU. Over time, they develop homeostasis disorders resulting in multiple organ failure and death. Mortality in CCI exceeds that of the majority of malignant neoplasms and functional dependence remains in most of survivors.

In the present review, the attempt is made to show the main links of CCI pathogenesis which, if acted upon, can prevent unfavorable outcome. The publications describing epidemiology of CCI, its outcomes, and clinical phenotype have been analyzed.

Several researchers consider CCI as a result of persistent inflammation, immunosuppression, and catabolism syndrome. Some works show the importance of nutrition for ICRU patients. The role of gastrointestinal tract in CCI formation has been noted. The effect of intensive therapy on microbiota of the ICRU patients has been demonstrated. Microbiome disturbances in dysbiosis and sepsis have been considered, as well as the effect of intestinal microbiome on the distant organs.

Post-intensive care syndrome is a significant constituent of CCI. The main sequelae of the syndrome, as well as the general questions of its prevention and treatment, have been denoted.

## Introduction

The term “chronic critical illness” (CCI) was introduced for the first time by Girard and Raffin [1] in 1985 when describing patients survived the acute phase of a disease but needed constant support and homeostasis correction under the condition of intensive therapy due to persistent organ dysfunction. However, precise universal criteria for the description of patients of this heterogenous group have not been worked out until present.

The CCI development is preceded with the emergence and interaction of several clinical syndromes determining the duration and severity of this state. The most important of them are systemic inflammatory response syndrome (SIRS); compensatory anti-inflammatory response syndrome (CARS); cytokine release syndrome (CRS); acute respiratory distress syndrome (ARDS), or multiple organ failure (MOF); persistent inflammation, immunosuppression, and catabolism syndrome (PICS). It is the PICS development that is considered the main pathophysiology of the chronic critical state or CCI [2]. The list of diseases underlying PICS with transformation into CCI is broad. Consciousness impairment is observed in the majority of patients at the stage of the marked clinical manifestations. This impairment is primarily associated with a toxic and metabolic effect on the brain, as well as with medications usually administered in intensive care and resuscitation units (ICRU). A special and probably the most common group is represented by the patients with marked alterations of consciousness having initial brain damage resulted from severe traumatic brain injury, ischemic or hemorrhagic stroke, or after neurosurgical operations [3]. CCI begins in this category of patients with the time of brain impairment with various traumatic agents (ischemia, hemorrhage, traumas, hypoxia, etc.). In severe brain injury, the secondary factors join the primary ones such as edema of the brain, its dislocation, and vascular complications: cerebral vasospasm or hyperemia [4].

Chronic critical illness is characterized by a long hospital stay, multiple organ disorders, high mortality rate, and considerable consumption of the resources [5]. Concurrently with protein-energy deficiency, patients experience significant alterations in metabolism, developing immunodeficiency, impairment of the gastrointestinal tract activity along with strongly reduced functional and cognitive capabilities. Additionally, inflammation persists for a long time, there are hormonal and neuromuscular disorders, the immunity is also weakened [2].

In order to maintain homeostasis, patients are subject to allostatic load (exhaustion) which in case of unfavorable course of the disease leads to multiple organ failure and fatal outcome [6].

In this review, the attempt was made to show the main links of CCI pathogenesis which, if acted upon, may prevent a poor outcome. Searching for the literature devoted to studying different aspects of CCI pathogenesis involved the following search engines: PubMed, Scopus, eLIBRARY.RU.

## Definition

Clinical CCI phenotype is described by various terms: neuropathy of critical illness, myopathy of critical illness, ICU-acquired weakness, and post-intensive care syndrome [7-10].

The variety of the terms is connected with a broad list of acute state diagnoses in patients. The completion of the acute stage is not followed by the improvement of the state, the disease transforms into a long-term multiple organ dysfunction which, in case of the unfavorable course, develops into sepsis and multiple organ failure [11, 12].

One of the earliest CCI criteria is a long period on MLV (21 successive days and more for 6 h a day and longer) [13].

Ischemic stroke, intracranial hemorrhages, tracheostoma, and sepsis are suggested as additional criteria [14-18].

Some authors [19, 20] suggest using the term “chronic critical illness” for patients undergoing intensive therapy over 7 days, who are observed to have organ dysfunction, nutrient insufficiency, muscular weakness, and reduction of cognitive capabilities during prolong hospitalization. Many of them do not achieve functional independence having the prognosis of poor long-term survival.

**Figure 1.**
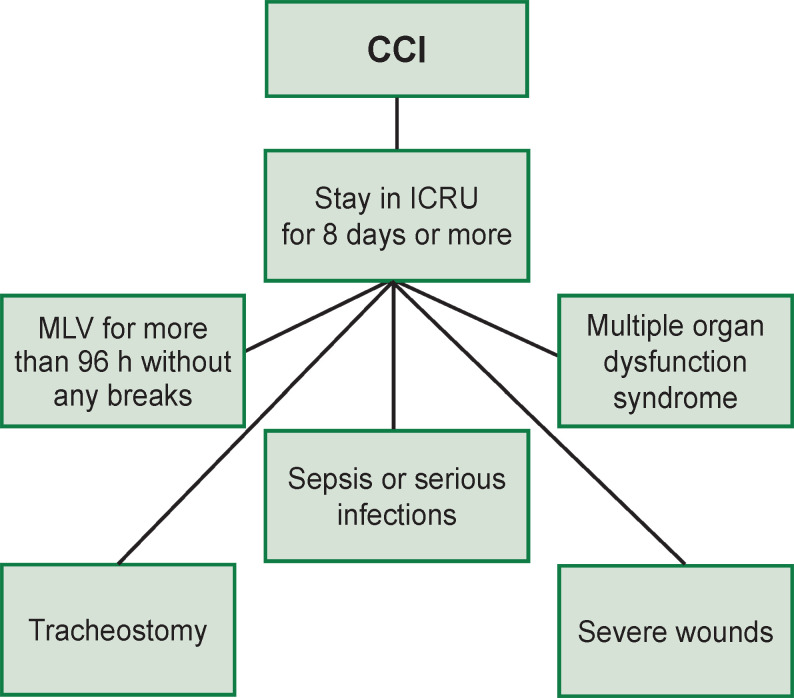
The main criteria of CCI development (according to the data of Research Triangle Institute [21])

Research Triangle Institute has defined the following criteria of CCI development (Figure 1): staying in the ICRU for 8 days or more and having one or more of the five states: MLV lasting over 96 h without breaks; tracheostomy; sepsis/severe infections; serious wounds, and multiple organ dysfunction syndrome (MODS) [21].

It should be taken into consideration that prolong stay in the ICRU results most likely in undesired problems since this is one of the factors of CCI formation.

## Epidemiology

The prevalence of CCI varies from 5 to 20% among ICRU patients [13]. This wide range may be accounted for by the absence of consensus regarding diagnostic criteria.

The majority of patients with CCI (>60%) are diagnosed with sepsis which is usually associated with MOF. Hospital mortality is about 30%, one-year survival is less than 50%, and only 10% return to functional independence [22, 23].

In the middle of 1980s, European researchers reported that MOF frequently occurs without any identified infection focus [24]. Later, SIRS was established to be caused both by infected and non-infected traumas. As a result, the main mechanisms of this phenomenon have been identified: bacterial translocation, cytokine storm, ischemia-reperfusion injury, etc. [25]. The epidemiological investigations have shown that the MOF syndrome has evolved into bimodal phenomenon with the decrease of the early mortality rate and increase of the late one [26-28].

The early MOF arises either after a primary severe trauma or after a secondary (nosocomial) infection [29].

Compensatory anti-inflammatory response syndrome was proposed for observation of SIRS and designed to explain an elevated susceptibility to infection and bimodal distribution of patients. Like SIRS, it represents a complicated and insufficiently defined pattern of immunological reactions in response to a severe infection. The difference is that SIRS is a proinflammatory syndrome which is directed to the elimination of the infectious organisms by activation of the immune system, while CARS, on the contrary, influences inactivation of the immune system and is directed to restoration of homeostasis from the inflammatory condition. Besides, CARS possesses a marked set of cytokines and cellular reactions and may exert a powerful effect on clinical outcomes in sepsis [30].

Modern diagnostic and intensive therapy modalities allow many patients to survive the acute phase of a disease. A significant part of patients with MOF survive after a long stay in the intensive care unit with a subsequent CCI development which is characterized by persistent inflammation, immunodepression, and catabolism [31]. The term “persistent inflammation, immunosuppression, and catabolism syndrome” was introduced for those patients who have survived primary sepsis/trauma but became chronically seriously ill. This syndrome was first described by Gentile et al. in 2012 [10] for a better understanding of pathophysiology of concurrently running processes of persistent inflammation (known as systemic inflammatory response syndrome), adaptive immunosuppression (known as compensatory anti-inflammatory response syndrome), and protein catabolism.

**Figure 2.**
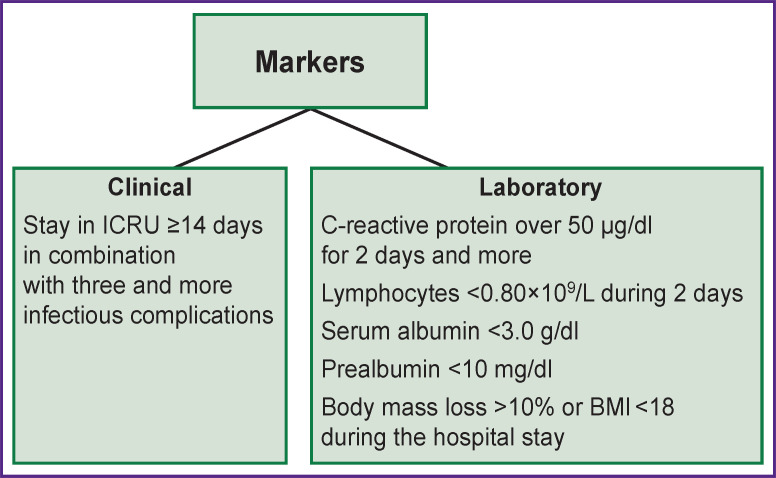
Markers for PICS identification

There are clinical and laboratory markers [20] which are used to identify PICS. Clinical markers include staying in ICRU for ≥14 days and ≥3 concomitant infectious complications. The level of C-reactive protein over 50 μg/dl for 2 or more days; immunosuppression characterized by a total amount of lymphocytes <0.80×109/L for 2 or fewer days; catabolism with serum albumin <3.0 g/dl, prealbumin <10 mg/dl, loss of body mass >10% or BMI <18 during hospital stay pertain to the laboratory markers (Figure 2).

Patients with PICS are admitted to the intensive care unit after a severe trauma or infection, have a significant early inflammatory and immunosuppressive response which leads to the ongoing organ damage, persistent inflammation, and immunity suppression with the loss of the muscular mass [32].

Under the normal physiological conditions, the immature myeloid cells (IMCs) are known to differentiate into granulocytes, monocytes/ macrophages, and dendrite cells, however, the inflammatory medium in a septic patient changes and maturation is impaired. In severe sepsis/septic shock, IL-6, IL-10, IL-12, dsRNA, IFN-γ, VEGF, G-CSF, GM-CSF, LPS, SCF, IL-1β, IL-13, IL-17, S100A8/9 prostoglandines, SAA, and CCL2 become involved in the cascade of the signaling molecules [33, 34]. IMCs, mainly granulocytes, possess strong suppressive properties, are proinflammatory cells and carriers of “bad” antigens.

The key factor of persistent inflammation and immunosuppression are myeloid-derived suppressor cells (MDSCs) [26]. They may impact actually every cell of the natural (or innate) and adaptive host immunity [35]. The reduction in the number of mature myeloid cells results in the considerable increase of MDSCs which act through several mechanisms contributing to inflammation and global inhibition of the adaptive immune function [35, 36].

The number of circulating MDSCs in patients with severe sepsis and septic shock is significantly elevated in the first 28 days from the beginning of sepsis. Primary MDSCs phenotype is granulocytic. During the first 24 h, patients with early death (<14 days) have much more MDSCs than patients with a favorable course of intensive therapy [32]. Fast decrease of MDSCs is observed in patients with early discharge from the intensive care unit [37, 38].

## Nutritional disorders and nutritive support in chronic critical illness

The damaged brain with its metabolic demands exceeding considerably those of other organs plays a crucial role in formation of protein-energy deficiency. It has been noted that nutrition insufficiency with increased metabolism in patients with a serious brain injury in ICRU is on average from 22 to 43% [39-41]. In this aspect, a nutritive support is an important constituent of the therapy. Besides, enteral nutrition helps maintain structural [42] and functional condition of the intestine including activation of T-cell association of lymphoid tissue [43] and activation of neutrophils [44]. Enteral nutrition allows for preservation of the mucous barrier which prevents translocation of bacteria from the intestinal lumen to the bloodstream and thereby hinders infection from spreading [45].

According to the ESPEN (European Society for Clinical Nutrition and Metabolism) guidelines, ICRU patients are indicated hypocaloric enteral/parenteral nutrition in the first 3 days not exceeding 70% of the energy demands. Caloric value should be increased up to 100% by day 7 [46]. It has been established [47, 48] that the increase of calories in the nutrition >70% during the first 12–24 h after the admission to ICRU does not lead to the reduction of hospital stay and better survival.

It should be noted that the volume of enteral/ parenteral nutrition in the USA makes up on average from 35 to 42% of the patient requirements in energy and protein which is considerably lower than is recommended by the ASPEN (American Society for Parenteral and Enteral Nutrition) and ESPEN [46, 49].

To maintain protein synthesis and homeostasis in the cells of critical patients, ASPEN recommend introduction of protein in the amount not less than 1.2 g/kg/day, ESPEN — 1.3 g/kg/day [46, 49].

At the same time, it is reported about intolerance of enteral feeding by critical patients in 30–75% of cases [50-53]. The most common symptoms of intolerance are vomiting, a large residual stomach volume, bloating, and diarrhea [50, 54].

The causes of malabsorption may be separate ingredients of the nutrient mixture, especially lipids. In contrast to the long-chain triglycerides, medium-chain triglycerides are directly absorbed into the portal circulation and do not require bile salts [55].

The protein origin and type can also affect intolerance. The protein hydrolyzed up to peptides requires less digestion reducing the risk of malabsorption [56].

## Gastrointestinal tract in the formation of chronic critical illness and multiple organ failure

Arterial hypotension of various genesis results in the impairment of gastrointestinal tract perfusion with consequent damage to the organs.

A serious brain injury is often accompanied by the development of arterial hypotension which may be caused by the reduction of systemic vascular resistance due to the damage to the diencephalic region, growing signs of brain dislocation, development of adrenal failure. One more cause of hypotension is the drop of cardiac output due to the decreased myocardial contractility or hypovolemia which may be the result of hemorrhage, dehydration therapy, diabetes insipidus, and hyperthermia. Hypovolemia initiates centralization of blood circulation which later may lead to a number of unfavorable consequences such as impairment of blood circulation in capillaries, ischemia of organs and tissues, tissue edema, and multiple organ failure [57].

Thus, already from the first hours after traumatic brain injury, the gastrointestinal tract experiences adverse effects induced by the circulation centralization and intestine ischemization.

The intestine consists of three interconnected components: epithelium, microbiota, and immune system. The intestine also contains over 80% of the total amount of lymphocytes in the organism [58].

The intestine is constantly regenerating owing to multipotent stem cells at the crypt base. They give rise to the four main types of the intestinal cells: a) enterocytes which absorb nourishing substances and make up >90% of the intestinal epithelial cells; b) mucus-producing caliciform cells; c) hormone-producing enteroendocrine cells; d) Paneth dephenzine-producing cells which protect intestinal stem cells and play a role in the interaction of the intestine with microbiota. A way from the generation, differentiation, and migration of the cells along the villi up to apoptosis or luminal delamination of the intact cells takes only 5–7 days in a healthy man [59].

In the last quarter of the 20^th^ century, a hypothesis was advanced that intestine is a motor of MODS [60].

The initial theories about the role of the intestine in critical illnesses suggested that hyperpermeability of the gut wall leads to translocation of bacteria in the systemic circulation with their subsequent spread via the vascular system. In reality, everything appeared to be more complicated. All intestinal elements — epithelium, immune system, and microbiome — are susceptible to critical illnesses and may, in their turn, cause a cascade of pathological reactions. Additionally, alterations in the intestine are capable of resulting in local and distant disorders through the changes in homeostatic processes and protective mechanisms and also in the release of toxic mediators into mesenteric lymph and systemic circulation [60].

It has been suggested that critical illness causes intestinal hyperpermeability which leads to translocation of intact bacteria into the blood flow with subsequent systemic manifestations [61].

Lymphatic system also links the intestine to the distant organs. Intestinal lymph flows out from the mesenteric lymph duct and ultimately joins the pulmonary circulation [62]. Numerous models of critical illnesses on animals have shown that ligation of the mesenteric lymph ducts reduces lung injury and neutrophil activation, and importantly, improves survival [63]. As a rule, the intestinal lymph does not contain bacteria, endotoxins, or cytokines [64]. Protein and lipid factors in the intestinal lymph are likely to stimulate Toll-like receptor 4 (TLR4) activating neutrophils in the lungs [65].

The specificity of lipid processing plays a certain role in transporting toxic lymph. Intestinal microsomal triglyceride transfer protein provides formation of hilomicrons in the lumen and absorption of lipids via the lymphatic system [66].

Intestinal mucus is the main barrier preventing digestive enzymes from reaching the epithelium and causing its destruction [67]. The destruction of the mucus protective barrier in shock or peritonitis may be inhibited with a tranexamic acid, aprotinin or 6-amedino-2-naphthyl p-guanidinobenzoate dimethanesulfonate (Nafamostat) for protease suppression. This process improves survival in preclinical models of the critical illness [68]. The protective functions of the mucus may help prevent autodigestion — the process destroying the wall of the intestine due to the presence of digestive enzymes in its lumen [69].

## Microbiota in patients with chronic critical illness

Human micribiota consists of more than 40 trillion bacteria, viruses, archaea, and fungi, most of which live in the gut [70-72]. Application of new methods of studying bacterial population enlarged the volume of actual data showing that microbiome is an important factor in the pathophysiology of a whole spectrum of diseases [73-77].

Microbiome protects the organism from infection, participates in drug metabolism, vitamin synthesis, nourishment. Impairment of microbiota homeostasis results in the development of intestine diseases, obesity, diabetes, and cardiovascular diseases. On the basis thereof, numerous disease may be prevented and even treated by acting upon microbiota [77].

Presently, microbiome is recognized a separate organ considering its diverse roles in metabolic processes, development of the immune system, protection from pathogens, and also involvement in the nutrient metabolism and preservation of the mucous barrier, in the work of the intestinal nervous system and motility [78-80].

The majority of intensive therapy patients receive antibiotics which are known to destroy microbiome [81]. They kill commensal microbiota, which leads to the likelihood of the secondary penetration of the pathogens and higher resistance to antibiotics [82]. There are numerous external modulators of intestinal microbiota other than antibiotics: different ways of eating; inhibition of gastric acid; intake of sedatives, opioids, and vasopressors [76, 83].

Intestinal microbiota depends largely on the presence of enteral nutrients while critical illness places it into the condition of acute starvation [84].

Besides, various interventions (for example, skin disinfection, treatment of the oral cavity) may change specific conditions of microbiota existence, and invasive procedures (endotracheal intubation, intravascular catheters) may impair natural barrier mechanisms facilitating penetration of microbes and their proliferation [85]. In this connection, interventions into microbiome are being developed to prevent and treat traumas and sepsis, e.g. application of probiotics, prebiotics, and synbiotics [77, 86].

### Change of microbial landscape

Patients admitted to the intensive care unit are observed to have dysbiosis of the intestinal microbiota [87, 88]. This microbiota in seriously ill patients is characterized by the less variety and amount of the key commensal genera (such as *Faecalibacterium prausnitzii*, *Blautia coccoides*, *Ruminococcus gnavus*), and in some cases by the increase (up to 50% and more of the total variety) of one genus, e.g. *Escherichia/Shigellа*, *Salmonella*, *Enterococcus*, *Clostridium difficile*, or *Staphylococcus* [89, 90].

The loss of microbiome variety is closely connected with the severity of patient’s condition. This underlines the clinical significance of intestinal microbiome in the intensive therapy of critical states [88]. Healthy intestinal microbiota protects against the invasion of pathogens such as *Enterococcus faecium*, *Escherichia coli*, and *C. difficile*. It is not surprising that severe infections caused by these pathogens are often encountered in patients recently receiving antibiotics. Their microbiota has probably been impaired, which led to the extreme growth of antibiotic-resistant and opportunistic bacteria [91].

Most pathogens do not act isolated therefore infections have “polymicrobial” phenotypes, and susceptibility to infections may be connected with the initial state of microbiota [92] and the severity of the infectious process [93].

### The effect of intestinal microbiome on distant organs

Recently, the hypothesis has been advanced that damage to the intestinal microbiome may lead to the damage in distant organs. The experimental studies on mice have shown the existence of the so-called axes: “intestine–lungs”, “intestine–brain”. Additionally to cytokines, communication in these axes is conceivably mediated by microbe-associated molecular patterns: lipopolysaccharides, peptidoglycan, and flagellin, as well as microbiota metabolites which are capable of translocation from the gut into systemic circulation with subsequent effect on the immune cells to enhance regulatory and proinflammatory responses [94, 95]. Thus, intestinal bacteria can direct the influx of the immune effector cells to the distant organs [96].

Patients with acute respiratory distress-syndrome have been noted to have a high content of the gut bacteria in the lung microbiomes, which correlates with a high content of the systemic inflammatory markers [97]. Other researchers have shown that systemic impact of microbiota-derived ligands increase the activity of alveolar macrophages and neutrophils of the bone marrow enhancing the elimination of gram-positive and gram-negative pathogens in the lungs [93, 98].

The connection between the gut microbiome and the brain is realized via numerous physiological channels including neuroendocrine and neuroimmune pathways and the vegetative nervous system [99]. Bacteria detected in the intestine are capable of creating neuromediators which can be found in the central nervous system [100]. For example, the *Lactobacillus brevis* strain may produce GABA [101].

Monoamines play a key role in the transmission of signals via the brain–intestine–microbiome axis [102]; they include serotonin and its precursor tryptophan [103], a key factor in the treatment of severe depression. Microbial products affecting the receptors of the human brain (intestine–brain axis) are responsible for encephalopathy in liver cirrhosis and delirium in the aged patients [104].

Such interactions between a distant organ and intestinal microbiome are being considered more and more often in the scientific literature as a theory of axes “intestine–organs” (“intestine–lungs”, “intestine– brain”, “intestine–kidneys”, and “intestine–liver”) [105].

## Post-intensive care syndrome

A common problem for all patients with CCI is a post-intensive care syndrome. In 2012, this term was recommended for the description of the new or worsening disorders in physical, cognitive, or psychic health condition arising after a critical illness and retaining after hospitalization in the acute period [106].

Patients with this syndrome may have the following problems acquired due to their stay in the intensive care unit: weakness caused by polyneuropathy and myopathy [107-109]; cachexia or exhaustion syndrome [110, 111]; organ dysfunction [112]; chronic pain [113]; sexual dysfunction [114, 115]; problems of psychic health including depression, anxiety, or post-traumatic stress disorder [116, 117]; neurocognitive disorders [118].

There are works reflecting the specificity of the post-intensive care syndrome in different fields of medicine: in oncology [119], pediatrics [120], geriatrics [121], and the assessment of its effect on the patient’s quality of life [122].

Clinical recommendations concerning the post-intensive care syndrome were issued under the aegis of the Federation of Anesthesiologists-Resuscitators of the Russian Federation, Society of Neuroanesthesiologists and Neuroresuscitators, and Association of Resuscitators of Russia in 2015. They determined the post-intensive syndrome as “a complex of somatic, neurological, social, and psychological consequences of staying in ICRU restricting patient’s everyday life” [122]. Cognitive, psychiatric, vegetative, neuromuscular, pulmonary complications, physical status, and quality of life were referred to the complication types of this syndrome. There has been noted a negative effect of a long-term bed-rest regiment on the most important body systems: musculoskeletal, respiratory, cardiovascular, metabolic, genitourinary, gastrointestinal, and nervous. Special significance is given to the immobilization syndrome — a complex of multiple organ disorders associated with a non-physiological restriction (the non-use phenomenon) of the patient’s motor and cognitive activity due to organic disorders of the CNS [123]. The immobilization syndrome forms orthostatic insufficiency, polyneuropathy of critical states (ICU-acquired weakness syndrome), and due to the weakness of the diaphragm and intercostal muscles makes the transition of the patient to the independent respiration difficult [124].

## Intensive therapy of chronic critical illness

One of the main reasons of a steady increase of patients with CCI is that problems at the stage of the acute phase were not solved effectively enough. The most important of them are: secondary brain injury in patients of the neurological and neurosurgical profile; protein-energy deficiency in presence of hypercatabolism/hypermetabolism; disturbance of the gastrointestinal activity in combination with microbiota impairment; immunity disorders; formation of comorbid complications leading to multiple organ failure [125, 126].

Treatment of this category of patients requires a complex multidisciplinary approach with the engagement of a large quantity of specialists and application of diverse methods of diagnosing, treatment, and a wide list of pharmaceuticals [127].

Prevention of CCI requires performance of the ABCDEFGH bundle [128-130] (ABCDE are the main and FGH are the additional components directed to the prevention of the post-intensive care syndrome):

A — airway management;

B — breathing trials: assessment of respiration including daily intervals in mechanical ventilation, revealing spontaneous awakening and occurrence of spontaneous breathing;

C — coordination of care and communication: choice of analgesia and sedation, coordination of care and communication;

D — delirium assessment: prevention and management;

E — early mobility and physical exercises;

F — family involvement and follow-up referrals, functional reconciliation;

G — good hand-off communication;

H — handout materials: available information materials.

According to the recommendations [122], the structure of measures for prevention and treatment of the post-intensive care syndrome incorporates the following:

1. Prevention of emotional and cognitive complications considering employment of analgosedation to prevent delirium; prevention of circadian rhythm disorders, and cognitive-afferent dissonance in ICRU patients.
2. Early mobilization as an element of the rehabilitation process for patients in ICRU. Of great importance is the prophylaxis of dysphagia as a factor of nutritive deficit as well as the choice of mobilization technique under the ICRU conditions. It is necessary to calculate the loads when planning mobilization. Of special significance are patient verticalization and the possibility to carry out rehabilitation of the MLV patients and monitoring of their state in the process of mobilization.
3. Socialization of ICRU patients during their sessions with an ergotherapeutist.

By the time of CCI formation along with the decreasing problems relating to the acute period of the main disease (the acute period for a traumatic brain injury is from 2 to 10 weeks depending on the clinical form [131]) the first place is being occupied by the successively arising comorbid conditions and diseases which considerably complicate the treatment and in the majority of cases are the main cause of lethal outcomes [132].

Correction of comorbid diseases is done in presence of chronic inflammation, disturbances in autoregulation of hemodynamics, trophism, immunity which are combined with the post-comatose consciousness disorders beginning with the level of unresponsive wakefulness (a vegetative status) [133, 134].

Many researchers report that early rehabilitation of the postoperative patients [135-137] is an effective method of CCI prevention and treatment.

## Conclusion

The question, put in 1985, whether attempts should be made to save chronic critically ill patients, remains urgent until now because of a low effectiveness of treatment measures and a high incidence of adverse outcomes

Serious scientific investigations are needed to study the pathogenesis of chronic critical illness and methods of its diagnosis, develop pathogenetically grounded intensive therapy and rehabilitation treatment, create the effective system to prevent the development of this condition.

## References

[1] Girard K., Raffin T.A. (1985). The chronically critically ill: to save or let die?. Respir Care.

[2] Hawkins R.B., Raymond S.L., Stortz J.A., Horiguchi H., Brakenridge S.C., Gardner A., Efron P.A., Bihorac A., Frederick M.S., Moore F.A., Moldawer L.L. (2018). Chronic critical illness and the persistent inflammation, immunosuppression, and catabolism syndrome.. Front Immunol.

[3] Piradov M.A., Suponeva N.A., Voznyuk I.A., Kondratyev A.N., Shchegolev A.V., Belkin A.A., Zaitsev O.S., Pryanikov I.V., Petrova M.V., Ivanova N.E., Gnedovskaya E.V., Ryabinkina Yu.V., Sergeev D.V., Iazeva E.G., Legostaeva L.A., Fufaeva E.V., Petrikov S.S. (2020). Russian Workgroup on Chronic Disorders of Consciousness. Chronic disorders of consciousness: terminology and diagnostic criteria. The results of the first meeting of the Russian Working Group for Chronic Disorders of Consciousness.. Annaly klinicheskoy i experimental’noy nevrologii.

[4] Potapov A.A., Krylov V.V., Gavrilov A.G., Kravchuk A.D., Likhterman L.B., Petrikov S.S., Talypov A.E., Zakharova N.E., Oshorov A.V., Sychev A.A., Alexandrova E.V., Solodov A.A. (2016). Guidelines for the diagnosis and treatment of severe traumatic brain injury. Part 2. Intensive care and neuromonitoring.. Voprosy nejrohirurgii im. N.N. Burdenko.

[5] Nelson J.E., Cox C.E., Hope A.A., Carson S.S. (2010). Chronic critical illness.. Am J Respir Crit Care Med.

[6] Loss S.H., Nunes D.S.L., Franzosi O.S., Salazar G.S., Teixeira C., Vieira S.R.R. (2017). Chronic critical illness: are we saving patients or creating victims?. Rev Bras Ter Intensiva.

[7] Shepherd S., Batra A., Lerner D.P. (2017). Review of critical illness myopathy and neuropathy.. Neurohospitalist.

[8] Tankisi H., de Carvalho M., Z’Graggen W.J. (2020). Critical illness neuropathy.. J Clin Neurophysiol.

[9] Kress J.P., Hall J.B. (2014). ICU-acquired weakness and recovery from critical illness.. N Engl J Med.

[10] Gentile L.F., Cuenca A.G., Efron P.A., Ang D., Bihorac A., McKinley B.A., Moldawer L.L., Moore F.A. (2012). Persistent inflammation and immunosuppression: a common syndrome and new horizon for surgical intensive care.. J Trauma Acute Care Surg.

[11] Mira J.C., Cuschieri J., Ozrazgat-Baslanti T., Wang Z., Ghita G.L., Loftus T.J., Stortz J.A., Raymond S.L., Lanz J.D., Hennessy L.V., Brumback B., Efron P.A., Baker H.V., Moore F.A., Maier R.V., Moldawer L.L., Brakenridge S.C. (2017). The epidemiology of chronic critical illness after severe traumatic injury at two level one trauma centers.. Crit Care Med.

[12] Tompkins R.G. (2015). Genomics of injury: the Glue Grant experience.. J Trauma Acute Care Surg.

[13] Loss S.H., Marchese C.B., Boniatti M.M., Wawrzeniak I.C., Oliveira R.P., Nunes L.N., Victorino J.A. (2013). Prediction of chronic critical illness in a general intensive care unit.. Rev Assoc Med Bras.

[14] Carson S.S. (2012). Definitions and epidemiology of the chronically critically ill.. Respir Care.

[15] Darvall J.N., Boonstra T., Norman J., Murphy D., Bailey M., Iwashyna T.J., Bagshaw S.M., Bellomo R. (2019). Persistent critical illness: baseline characteristics, intensive care course, and cause of death.. Crit Care Resusc.

[16] Bagshaw S.M., Stelfox H.T., Iwashyna T.J., Bellomo R., Zuege D., Wang X. (2018). Timing of onset of persistent critical illness: a multi-centre retrospective cohort study.. Intensive Care Med.

[17] Sjoding M.W., Cooke C.R. (2015). Chronic critical illness: a growing legacy of successful advances in critical care.. Crit Care Med.

[18] Loss S.H., Oliveira R.P., Maccari J.G., Savi A., Boniatti M.M., Hetzel M.P., Dallegrave D.M., Balzano Pde C., Oliveira E.S., Höher J.A., Torelly A.P., Teixeira C. (2015). The reality of patients requiring prolonged mechanical ventilation: a multicenter study.. Rev Bras Ter Intensiva.

[19] Lamas D. (2014). Chronic critical illness.. N Engl J Med.

[20] Mira J.C., Brakenridge S.C., Moldawer L.L., Moore F.A. (2017). Persistent inflammation, immunosuppression and catabolism syndrome.. Crit Care Clin.

[21] Kandilov A.M., Ingber M., Morley M., Coomer N.M., Dalton K., Gage B., Superina C., Kennell D. (2014). Chronically Critically Ill Population Payment Recommendations (CCIP-PR)..

[22] Marchioni A., Fantini R., Antenora F., Clini E., Fabbri L. (2015). Chronic critical illness: the price of survival.. Eur J Clin Invest.

[23] Kahn J.M., Le T., Angus D.C., Cox C.E., Hough C.L., White D.B., Yende S., Carson S.S. (2015). ProVent Study Group Investigators. The epidemiology of chronic critical illness in the United States.. Crit Care Med.

[24] Goris R.J., te Boekhorst T.P., Nuytinck J.K., Gimbrère J.S. (1985). Multiple-organ failure. Generalized autodestructive inflammation?. Arch Surg.

[25] Chakraborty R.K., Burns B. (2022). Systemic inflammatory response syndrome 2021.. StatPearls..

[26] Efron P.A., Mohr A.M., Bihorac A., Horiguchi H., Hollen M.K., Segal M.S., Baker H.V., Leeuwenburgh C., Moldawer L.L., Moore F.A., Brakenridge S.C. (2018). Persistent inflammation, immunosuppression, and catabolism and the development of chronic critical illness after surgery.. Surgery.

[27] Dewar D.C., Tarrant S.M., King K.L., Balogh Z.J. (2013). Changes in the epidemiology and prediction of multiple-organ failure after injury.. J Trauma Acute Care Surg.

[28] Sauaia A., Moore E.E., Johnson J.L., Chin T.L., Banerjee A., Sperry J.L., Maier R.V., Burlew C.C. (2014). Temporal trends of postinjury multiple-organ failure: still resource intensive, morbid, and lethal.. J Trauma Acute Care Surg.

[29] Moore F.A., Sauaia A., Moore E.E., Haenel J.B., Burch J.M., Lezotte D.C. (1996). Postinjury multiple organ failure: a bimodal phenomenon.. J Trauma.

[30] Ward N.S., Casserly B., Ayala A. (2008). The compensatory anti-inflammatory response syndrome (CARS) in critically ill patients.. Clin Chest Med.

[31] Rosenthal M.D., Kamel A.Y., Rosenthal C.M., Brakenridge S., Croft C.A., Moore F.A. (2018). Chronic critical illness: application of what we know.. Nutr Clin Pract.

[32] Mira J.C., Gentile L.F., Mathias B.J., Efron P.A., Brakenridge S.C., Mohr A.M., Moore F.A., Moldawer L.L. (2017). Sepsis pathophysiology, chronic critical illness, and persistent inflammation-immunosuppression and catabolism syndrome.. Crit Care Med.

[33] Gabrilovich D.I., Nagaraj S. (2009). Myeloid-derived suppressor cells as regulators of the immune system.. Nat Rev Immunol.

[34] Dilek N., Vuillefroy de Silly R., Blancho G., Vanhove B. (2012). Myeloid-derived suppressor cells: mechanisms of action and recent advances in their role in transplant tolerance.. Front Immunol.

[35] Zhou H., Jiang M., Yuan H., Ni W., Tai G. (2021). Dual roles of myeloid-derived suppressor cells induced by Toll-like receptor signaling in cancer.. Oncol Lett.

[36] Chun E., Lavoie S., Michaud M., Gallini C.A., Kim J., Soucy G., Odze R., Glickman J.N., Garrett W.S. (2015). CCL2 promotes colorectal carcinogenesis by enhancing polymorphonuclear myeloid-derived suppressor cell population and function.. Cell Rep.

[37] Janols H., Bergenfelz C., Allaoui R., Larsson A.M., Rydén L., Björnsson S., Janciauskiene S., Wullt M., Bredberg A., Leandersson K. (2014). A high frequency of MDSCs in sepsis patients, with the granulocytic subtype dominating in gram-positive cases.. J Leukoc Biol.

[38] Mathias B., Delmas A.L., Ozrazgat-Baslanti T., Vanzant E.L., Szpila B.E., Mohr A.M., Moore F.A., Brakenridge S.C., Brumback B.A., Moldawer L.L., Efron P.A. (2017). the Sepsis, Critical Illness Research Center Investigators. Human myeloid-derived suppressor cells are associated with chronic immune suppression after severe sepsis/septic shock.. Ann Surg.

[39] Imoberdorf R., Meier R., Krebs P., Hangartner P.J., Hess B., Stäubli M., Wegmann D., Rühlin M., Ballmer P.E. (2010). Prevalence of undernutrition on admission to Swiss hospitals.. Clin Nutr.

[40] Pirlich M., Schütz T., Kemps M., Luhman N., Minko N., Lübke H.J., Rossnagel K., Willich S.N., Lochs H. (2005). Social risk factors for hospital malnutrition.. Nutrition.

[41] Marinho R., Pessoa A., Lopes M., Rosinhas J., Pinho J., Silveira J., Amado A., Silva S., Oliveira B.M.P.M., Marinho A., Jager-Wittenaar H. (2020). High prevalence of malnutrition in Internal Medicine wards — a multicentre ANUMEDI study.. J Intern Med.

[42] Groos S., Hunefeld G., Luciano L. (1996). Parenteral versus enteral nutrition: morphological changes in human adult intestinal mucosa.. J Submicrosc Cytol Pathol.

[43] Yamamoto S., Allen K., Jones K.R., Cohen S.S., Reyes K., Huhmann M.B. (2020). Meeting calorie and protein needs in the critical care unit: a prospective observational pilot study.. Nutr Metab Insights.

[44] Meyer J., Yurt R.W., Duhaney R., Hesse D.G., Tracey K.J., Fong Y., Richardson D., Calvano S., Dineen P., Shires G.T. (1988). Differential neutrophil activation before and after endotoxin infusion in enterally versus parenterally fed volunteers.. Surg Gynecol Obstet.

[45] Jung C.Y., Bae J.M. (2021). Pathophysiology and protective approaches of gut injury in critical illness.. Yeungnam Univ J Med.

[46] Singer P., Blaser A.R., Berger M.M., Alhazzani W., Calder P.C., Casaer M.P., Hiesmayr M., Mayer K., Montejo J.C., Pichard C., Preiser J.C., van Zanten A.R.H., Oczkowski S., Szczeklik W., Bischoff S.C. (2019). ESPEN guideline on clinical nutrition in the intensive care unit.. Clin Nutr.

[47] Chapman M., Peake S.L., Bellomo R., Davies A., Deane A., Horowitz M., Hurford S., Lange K., Little L., Mackle D., O’Connor S., Presneill J., Ridley E., Williams P., Young P. (2018). TARGET Investigators, for the ANZICS Clinical Trials Group. Energy-dense versus routine enteral nutrition in the critically ill.. N Eng J Med.

[48] Yamamoto S., Allen K., Jones K.R., Cohen S.S., Reyes K., Huhmann M.B. (2020). Meeting calorie and protein needs in the critical care unit: a prospective observational pilot study.. Nutr Metab Insights.

[49] McClave S.A., Taylor B.E., Martindale R.G., Warren M.M., Johnson D.R., Braunschweig C., McCarthy M.S., Davanos E., Rice T.W., Cresci G.A., Gervasio J.M., Sacks G.S., Roberts P.R., Compher C. (2016). Society of Critical Care Medicine; American Society of Parenteral and Enteral Nutrition. Guidelines for the provision and assessment of nutrition support therapy in the adult critically ill patient: Society of Critical Care Medicine (SCCM) and American Society for Parenteral and Enteral Nutrition (A.S.P.E.N.).. JPEN J Parenter Enteral Nutr.

[50] Gungabissoon U., Hacquoil K., Bains C., Irizarry M., Dukes G., Williamson R., Deane A.M., Heyland D.K. (2015). Prevalence, risk factors, clinical consequences, and treatment of enteral feed intolerance during critical illness.. JPEN J Parenter Enteral Nutr.

[51] Blaser A.R., Starkopf J., Kirsimägi Ü., Deane A.M. (2014). Definition, prevalence, and outcome of feeding intolerance in intensive care: a systematic review and meta-analysis.. Acta Anaesthesiol Scand.

[52] Atasever A.G., Ozcan P.E., Kasali K., Abdullah T., Orhun G., Senturk E. (2018). The frequency, risk factors, and complications of gastrointestinal dysfunction during enteral nutrition in critically ill patients.. Ther Clin Risk Manag.

[53] Merchan C., Altshuler D., Aberle C., Papadopoulos J., Schwartz D. (2017). Tolerability of enteral nutrition in mechanically ventilated patients with septic shock who require vasopressors.. J Intensive Care Med.

[54] Faramarzi E., Mahmoodpoor A., Hamishehkar H., Shadvar K., Iranpour A., Sabzevari T., Sanaie S. (2020). Effect of gastric residual volume monitoring on incidence of ventilator-associated pneumonia in mechanically ventilated patients admitted to intensive care unit.. Pak J Med Sci.

[55] Avgerinos K.I., Egan J.M., Mattson M.P., Kapogiannis D. (2020). Medium chain triglycerides induce mild ketosis and may improve cognition in Alzheimer’s disease. A systematic review and meta-analysis of human studies.. Ageing Res Rev.

[56] Mentec H., Dupont H., Bocchetti M., Cani P., Ponche F., Bleichner G. (2001). Upper digestive intolerance during enteral nutrition in critically ill patients: frequency, risk factors, and complications.. Crit Care Med.

[57] Rzheutskaya R.E. (2012). Characteristics of hemodynamic disorders in patients with severe traumatic brain injury.. Crit Care Res Pract.

[58] Thome J.J., Yudanin N., Ohmura Y., Kubota M., Grinshpun B., Sathaliyawala T., Kato T., Lerner H., Shen Y., Farber D.L. (2014). Spatial map of human T cell compartmentalization and maintenance over decades of life.. Cell.

[59] Clevers H.C., Bevins C.L. (2013). Paneth cells: maestros of the small intestinal crypts.. Annu Rev Physiol.

[60] Meng M., Klingensmith N.J., Coopersmith C.M. (2017). New insights into the gut as the driver of critical illness and organ failure.. Curr Opin Crit Care.

[61] Nagpal R., Yadav H. (2017). Bacterial translocation from the gut to the distant organs: an overview.. Ann Nutr Metab.

[62] Deitch E.A. (2012). Gut-origin sepsis: evolution of a concept.. Surgeon.

[63] Senthil M., Brown M., Xu D.Z., Lu Q., Feketeova E., Deitch E.A. (2006). Gut-lymph hypothesis of systemic inflammatory response syndrome/multiple-organ dysfunction syndrome: validating studies in a porcine model.. J Trauma.

[64] Reino D.C., Pisarenko V., Palange D., Doucet D., Bonitz R.P., Lu Q., Colorado I., Sheth S.U., Chandler B., Kannan K.B., Ramanathan M., Xu D.Z., Deitch E.A., Feinman R. (2011). Trauma hemorrhagic shock-induced lung injury involves a gut-lymph-induced TLR4 pathway in mice.. PLoS One.

[65] Reino D.C., Palange D., Feketeova E., Bonitz R.P., Xu D.Z., Lu Q., Sheth S.U., Peña G., Ulloa L., De Maio A., Feinman R., Deitch E.A. (2012). Activation of toll-like receptor 4 is necessary for trauma hemorrhagic shock-induced gut injury and polymorphonuclear neutrophil priming.. Shock.

[66] Xie Y., Newberry E.P., Young S.G., Robine S., Hamilton R.L., Wong J.S., Luo J., Kennedy S., Davidson N.O (2006). Compensatory increase in hepatic lipogenesis in mice with conditional intestine-specific Mttp deficiency.. J Biol Chem.

[67] Chang M., Alsaigh T., Kistler E.B., Schmid-Schönbein G.W. (2012). Breakdown of mucin as barrier to digestive enzymes in the ischemic rat small intestine.. PLoS One.

[68] DeLano F.A., Hoyt D.B., Schmid-Schönbein G.W. (2013). Pancreatic digestive enzyme blockade in the intestine increases survival after experimental shock.. Sci Transl Med.

[69] Schmid-Schönbein G.W., DeLano F.A., Penn A.H., Kistler E. (2012). An elementary analysis of physiologic shock and multi-organ failure: the autodigestion hypothesis.. Annu Int Conf IEEE Eng Med Biol Soc.

[70] Sender R., Fuchs S., Milo R. (2016). Are we really vastly outnumbered? Revisiting the ratio of bacterial to host cells in humans.. Cell.

[71] Liu S., Zhao W., Lan P., Mou X. (2021). The microbiome in inflammatory bowel diseases: from pathogenesis to therapy.. Protein Cell.

[72] Oami T., Chihade D.B., Coopersmith C.M. (2019). The microbiome and nutrition in critical illness.. Curr Opin Crit Care.

[73] Nakov R., Segal J.P., Settanni C.R., Bibbò S., Gasbarrini A., Cammarota G., Ianiro G. (2020). Microbiome: what intensivists should know.. Minerva Anestesiol.

[74] Dickson R.P. (2016). The microbiome and critical illness.. Lancet Respir Med.

[75] Otani S., Chihade D.B., Coopersmith C.M. (2018). Critical illness and the role of the microbiome.. Acute Med Surg.

[76] Haak B.W., Wiersinga W.J. (2017). The role of the gut microbiota in sepsis.. Lancet Gastroenterol Hepatol.

[77] Panigrahi P., Parida S., Nanda N.C., Satpathy R., Pradhan L., Chandel D.S., Baccaglini L., Mohapatra A., Mohapatra S.S., Misra P.R., Chaudhry R., Chen H.H., Johnson J.A., Morris J.G., Paneth N., Gewolb I.H. (2017). A randomized synbiotic trial to prevent sepsis among infants in rural India.. Nature.

[78] Barko P.C., McMichael M.A., Swanson K.S., Williams D.A. (2018). The gastrointestinal microbiome: a review.. Vet Intern Med.

[79] Weiss G.A., Hennet T. (2017). Mechanisms and consequences of intestinal dysbiosis.. Cell Mol Life Sci.

[80] Nicholson J.K., Holmes E., Kinross J., Burcelin R., Gibson G., Jia W., Pettersson S. (2012). Host-gut microbiota metabolic interactions.. Science.

[81] Vincent J.L., Rello J., Marshall J., Silva E., Anzueto A., Martin C.D., Moreno R., Lipman J., Gomersall C., Sakr Y., Reinhart K. (2009). EPIC II Group of Investigators. International study of the prevalence and outcomes of infection in intensive care units.. JAMA.

[82] Iizumi T., Battaglia T., Ruiz V., Perez Perez G.I. (2017). Gut microbiome and antibiotics.. Arch Med Res.

[83] Haak B.W., Levi M., Wiersinga W.J. (2017). Microbiota-targeted therapies on the intensive care unit.. Curr Opin Crit Care.

[84] Morowitz M.J., Carlisle E.M., Alverdy J.C. (2011). Contributions of intestinal bacteria to nutrition and metabolism in the critically ill.. Surg Clin North Am.

[85] Kitsios G.D., Morowitz M.J., Dickson R.P., Huffnagle G.B., McVerry B.J., Morris A. (2017). Dysbiosis in the ICU: microbiome science coming to the bedside.. J Crit Care.

[86] Kasatpibal N., Whitney J.D., Saokaew S., Kengkla K., Heitkemper M.M., Apisarnthanarak A. (2017). Effectiveness of probiotic, prebiotic, and synbiotic therapies in reducing postoperative complications: a systematic review and network meta-analysis.. Clin Infect Dis.

[87] Lankelma J.M., van Vught L.A., Belzer C., Schultz M.J., van der Poll T., de Vos W.M., Wiersinga W.J. (2017). Critically ill patients demonstrate large interpersonal variation in intestinal microbiota dysregulation: a pilot study.. Intensive Care Med.

[88] Moron R., Galvez J., Colmenero M., Anderson P., Cabeza J., Rodriguez-Cabezas M.E. (2019). The importance of the microbiome in critically ill patients: role of nutrition.. Nutrients.

[89] Zaborin A., Smith D., Garfield K., Quensen J., Shakhsheer B., Kade M., Tirrell M., Tiedje J., Gilbert J.A., Zaborina O., Alverdy J.C. (2014). Membership and behavior of ultra-low-diversity pathogen communities present in the gut of humans during prolonged critical illness.. mBio.

[90] Ojima M., Motooka D., Shimizu K., Gotoh K., Shintani A., Yoshiya K., Nakamura S., Ogura H., Iida T., Shimazu T. (2016). Metagenomic analysis reveals dynamic changes of whole gut microbiota in the acute phase of intensive care unit patients.. Dig Dis Sci.

[91] Pamer E.G. (2016). Resurrecting the intestinal microbiota to combat antibiotic-resistant pathogens.. Science.

[92] Hojo M., Asahara T., Nagahara A., Takeda T., Matsumoto K., Ueyama H., Matsumoto K., Asaoka D., Takahashi T., Nomoto K., Yamashiro Y., Watanabe S. (2018). Gut microbiota composition before and after use of proton pump inhibitors.. Dig Dis Sci.

[93] Schuijt T.J., Lankelma J.M., Scicluna B.P., de Sousa e Melo F., Roelofs J.J., de Boer J.D., Hoogendijk A.J., de Beer R., de Vos A., Belzer C., de Vos W.M., van der Poll T., Wiersinga W.J. (2016). The gut microbiota plays a protective role in the host defence against pneumococcal pneumonia.. Gut.

[94] Schuijt T.J., van der Poll T., de Vos W.M., Wiersinga W.J. (2013). The intestinal microbiota and host immune interactions in the critically ill.. Trends Microbiol.

[95] Budden K.F., Gellatly S.L., Wood D.L., Cooper M.A., Morrison M. (2017). Emerging pathogenic links between microbiota and the gut-lung axis.. Nat Rev Microbiol.

[96] Gray J., Oehrle K., Worthen G., Alenghat T., Whitsett J., Deshmukh H. (2017). Intestinal commensal bacteria mediate lung mucosal immunity and promote resistance of newborn mice to infection.. Sci Transl Med.

[97] Siwicka-Gieroba D., Czarko-Wicha K. Lung microbiome — a modern knowledge. (2020). Cent Eur J Immunol.

[98] Lankelma J.M., Birnie E., Weehuizen T.A.F., Scicluna B.P., Belzer C., Houtkooper R.H., Roelofs J.J.T.H., de Vos A.F., van der Poll T., Budding A.E., Wiersinga W.J. (2017). The gut microbiota as a modulator of innate immunity during melioidosis.. PLoS Negl Trop Dis.

[99] Clarke G., Stilling R.M., Kennedy P.J., Stanton C., Cryan J.F., Dinan T.G. (2014). Minireview: gut microbiota: the neglected endocrine organ.. Mol Endocrinol.

[100] Wall R., Cryan J.F., Ross R.P., Fitzgerald G.F., Dinan T.G., Stanton C., Lyte M., Cryan J.F. (2014). Bacterial neuroactive compounds produced by psychobiotics.. Microbial endocrinology: the microbiota-gut-brain axis in health and disease..

[101] Barrett E., Ross R.P., O’Toole P.W., Fitzgerald G.F., Stanton C. (2012). γ-Aminobutyric acid production by culturable bacteria from the human intestine.. J Appl Microbiol.

[102] Lyte M. (2013). Microbial endocrinology in the microbiome-gut-brain axis: how bacterial production and utilization of neurochemicals influence behavior.. PLoS Pathog.

[103] O’Mahony S.M., Clarke G., Borre Y.E., Dinan T.G., Cryan J.F. (2015). Serotonin, tryptophan metabolism and the brain-gut-microbiome axis.. Behav Brain Res.

[104] Bajaj J.S. (2014). The role of microbiota in hepatic encephalopathy.. Gut Microbes.

[105] Jacobs M.C., Haak B.W., Hugenholtz F., Wiersinga W.J. (2017). Gut microbiota and host defense in critical illness.. Curr Opin Crit Care.

[106] Needham D.M., Davidson J., Cohen H., Hopkins R.O., Weinert C., Wunsch H., Zawistowski C., Bemis-Dougherty A., Berney S.C., Bienvenu O.J., Brady S.L., Brodsky M.B., Denehy L., Elliott D., Flatley C., Harabin A.L., Jones C., Louis D., Meltzer W., Muldoon S.R., Palmer J.B., Perme C., Robinson M., Schmidt D.M., Scruth E., Spill G.R., Storey C.P., Render M., Votto J., Harvey M.A. (2012). Improving long-term outcomes after discharge from intensive care unit: report from a stakeholders’ conference.. Crit Care Med.

[107] Rengel K.F., Hayhurst C.J., Pandharipande P.P., Hughes C.G. (2019). Long-term cognitive and functional impairments after critical illness.. Anesth Analg.

[108] Latronico N., Bolton C.F. (2011). Critical illness polyneuropathy and myopathy: a major cause of muscle weakness and paralysis.. Lancet Neurol.

[109] Macht M., Wimbish T., Clark B.J., Benson A.B., Burnham E.L., Williams A., Moss M. (2011). Post-extubation dysphagia is persistent and associated with poor outcomes in survivors of critical illness.. Crit Care.

[110] Abunnaja S., Cuviello A., Sanchez J.A. (2013). Enteral and parenteral nutrition in the perioperative period: state of the art.. Nutrients.

[111] Reid C.L., Campbell I.T., Little R.A. (2004). Muscle wasting and energy balance in critical illness.. Clin Nutr.

[112] Opal S.M. (2011). Immunologic alterations and the pathogenesis of organ failure in the ICU.. Semin Respir Crit Care Med.

[113] Kemp H.I., Laycock H., Costello A., Brett S.J. (2019). Chronic pain in critical care survivors: a narrative review.. Br J Anaesth.

[114] Griffiths J., Gager M., Alder N., Fawcett D., Waldmann C., Quinlan J. (2006). A self-report-based study of the incidence and associations of sexual dysfunction in survivors of intensive care treatment.. Intensive Care Med.

[115] Ulvik A., Kvåle R., Wentzel-Larsen T., Flaatten H. (2008). Sexual function in ICU survivors more than 3 years after major trauma.. Intensive Care Med.

[116] Myhren H., Ekeberg O., Tøien K., Karlsson S., Stokland O. (2010). Posttraumatic stress, anxiety and depression symptoms in patients during the first year post intensive care unit discharge.. Crit Care.

[117] Winters B.D., Eberlein M., Leung J., Needham D.M., Pronovost P.J., Sevransky J.E. (2010). Long-term mortality and quality of life in sepsis: a systematic review.. Crit Care Med.

[118] Torgersen J., Hole J.F., Kvåle R., Wentzel-Larsen T., Flaatten H. (2011). Cognitive impairments after critical illness.. Acta Anaesthesiol Scand.

[119] Gunderson C.C., Walter A.C., Ruskin R., Ding K., Moore K.N. (2016). Post-intensive care unit syndrome in gynecologic oncology patients.. Support Care Cancer.

[120] Manning J.C., Pinto N.P., Rennick J.E., Colville G., Curley M.A.Q. (2018). Conceptualizing post intensive care syndrome in children — the PICS-p framework.. Pediatr Crit Care Med.

[121] Wang S., Allen D., Kheir Y.N., Campbell N., Khan B. (2018). Aging and post-intensive care syndrome: a critical need for geriatric psychiatry.. Am J Geriatr Psychiatry.

[122] Federation of Anesthesiologists-Resuscitators of Russia. Association of Neuroanesthesiologists and Neuroresuscitators. (2015). Union of Rehabilitologists of Russia.. Reabilitatsiya v intensivnoy terapii (ReabIT). Klinicheskie rekomendatsii [Rehabilitation in intensive care (ReabIT). Clinical guidelines]..

[123] Belkin A.A., Davyidova N.S., Leyderman I.N., Borovskikh S.V., Khalin A.V. (2014). Bed-rest in intensive care and resuscitation.. Meditsina-Ural.

[124] Belkin A.A. (2018). Syndrome effects of intensive therapy — post intensive care syndrome (PICS).. Vestnik intensivnoj terapii im. A.I. Saltanova.

[125] Xing C., Arai K., Lo E.H., Hommel M. (2012). Pathophysiologic cascades in ischemic stroke.. Int J Stroke.

[126] Sharma R., Shultz S.R., Robinson M.J., Belli A., Hibbs M.L., O’Brien T.J., Semple B.D. (2019). Infections after a traumatic brain injury: the complex interplay between the immune and neurological systems.. Brain Behav Immun.

[127] Marehbian J., Muehlschlegel S., Edlow B.L., Hinson H.E., Hwang D.Y. (2017). Medical management of the severe traumatic brain injury patient.. Neurocrit Care.

[128] Harvey M.A., Davidson J.E. (2016). Postintensive care syndrome: right care, right now… and later.. Crit Care Med.

[129] Inoue S., Hatakeyama J., Kondo Y., Hifumi T., Sakuramoto H., Kawasaki T., Taito S., Nakamura K., Unoki T., Kawai Y., Kenmotsu Y., Saito M., Yamakawa K., Nishida O. (2019). Post-intensive care syndrome: its pathophysiology, prevention, and future directions.. Acute Med Surg.

[130] Ely E.W. The ABCDEF bundle: science and philosophy of how ICU liberation serves patients and families.. Crit Care Med.

[131] Likhterman L.B. (1990). Principles of modern periodization of the course of traumatic brain injury.. Voprosy nejrohirurgii im. N.N. Burdenko.

[132] Parfenov A.L., Petrova M.V., Pichugina I.M., Luginina E.V. (2020). Comorbidity development in patients with severe brain injury resulting in chronic critical condition (review).. Obsaa reanimatologia.

[133] Iwashyna T.J., Hodgson C.L., Pilcher D., Orford N., Santamaria J.D., Bailey M., Bellomo R. (2015). Towards defining persistent critical illness and other varieties of chronic critical illness.. Crit Care Resusc.

[134] Iwashyna T.J., Hodgson C.L., Pilcher D., Bailey M., Bellomo R. (2015). Persistent critical illness characterised by Australian and New Zealand ICU clinicians.. Crit Care Resusc.

[135] Chiung-Jui Su D., Yuan K.S., Weng S.F., Hong R.B., Wu M.P., Wu H.M., Chou W. (2015). Can early rehabilitation after total hip arthroplasty reduce its major complications and medical expenses? Report from a nationally representative cohort.. Biomed Res Int.

[136] Bugbee W.D., Pulido P.A., Goldberg T., D’Lima D.D. (2016). Use of an anti-gravity treadmill for early postoperative rehabilitation after total knee replacement: a pilot study to determine safety and feasibility.. Am J Orthop (Belle Mead NJ).

[137] Michot A., Stoeckle E., Bannel J.D., Colombani S., Sargos P., Brouste V., Italiano A., Kind M. (2015). The introduction of early patient rehabilitation in surgery of soft tissue sarcoma and its impact on post-operative outcome.. Eur J Surg Oncol.

